# Supplementary figures and images for: Cartography of teneurin and latrophilin expression reveals spatiotemporal axis heterogeneity in the mouse hippocampus during development
Source: PLoS Biol. 2024 May 7;22(5):e3002599. doi: 10.1371/journal.pbio.3002599 (PMC11101112; doi:10.1371/journal.pbio.3002599)

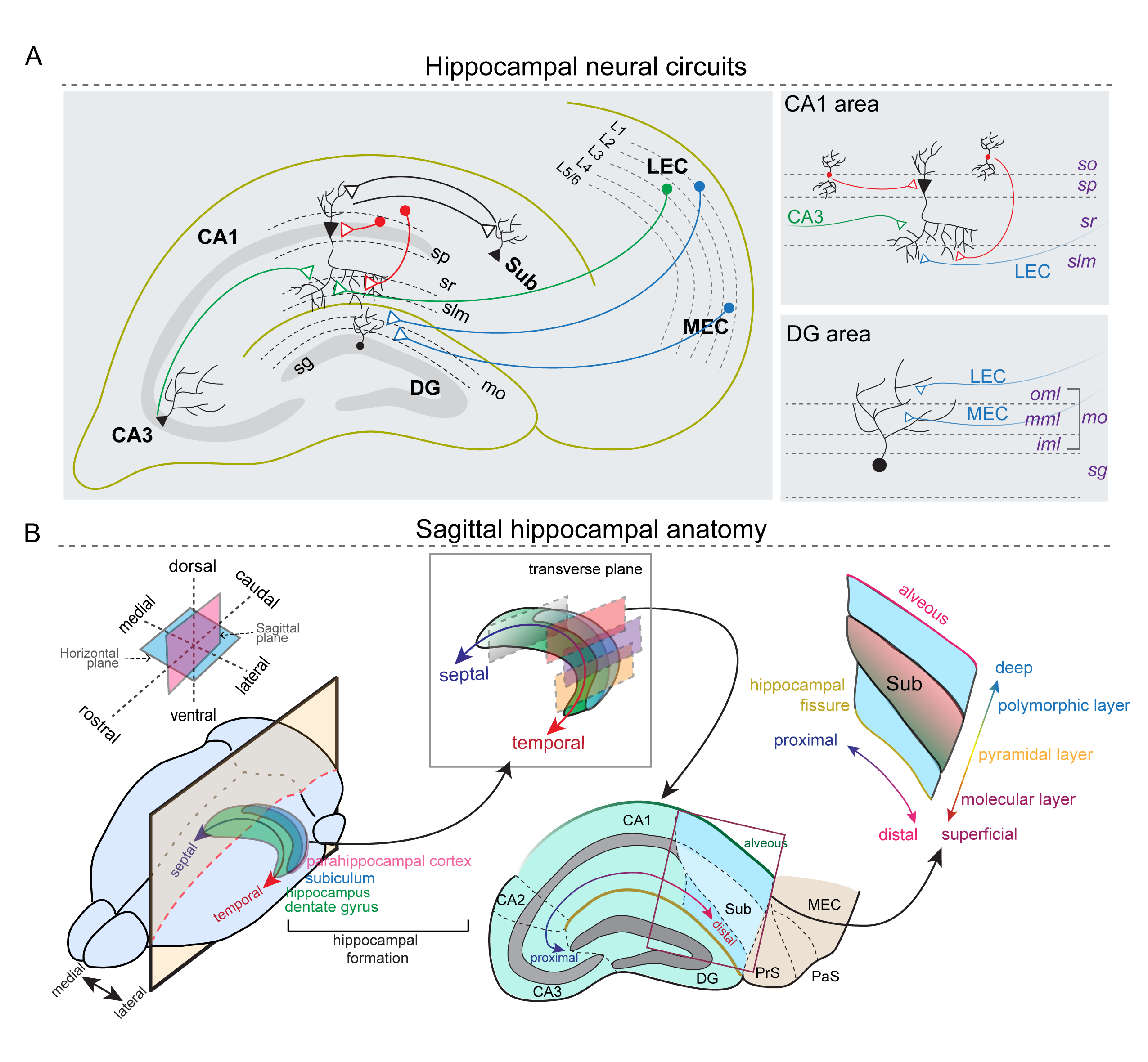

Supplement: S1 Fig — (A) Schematic diagram shows adult mouse hippocampal neural circuits with cells of CA1, CA3, DG, lateral, and medial entorhinal (LEC and MEC) areas. (B) Schematic diagram of hippocampus anatomy visualized through sagittal view for a spatial orientation. Transverse section of hippocampal region is shown to highlight the layers within the CA1 and subiculum regions. so–stratum oriens; sp–stratum pyramidale; sr–stratum radiatum; slm–stratum lacunosum-moleculare; mo–molecular layer; oml–outer molecular layer; mml–middle molecular layer; iml–inner molecular layer; sg–stratum granulare; po–polymorph layer; Sub–subiculum; PaS–parasubiculum; PrS–presubiculum; L1-L6 –layer 1–6; CA1, CA3 –cornu Ammonis 1, 3; DG–dentate gyrus; LEC and MEC–lateral and medial entorhinal cortex. (TIF) [file pbio.3002599.s001.tif]

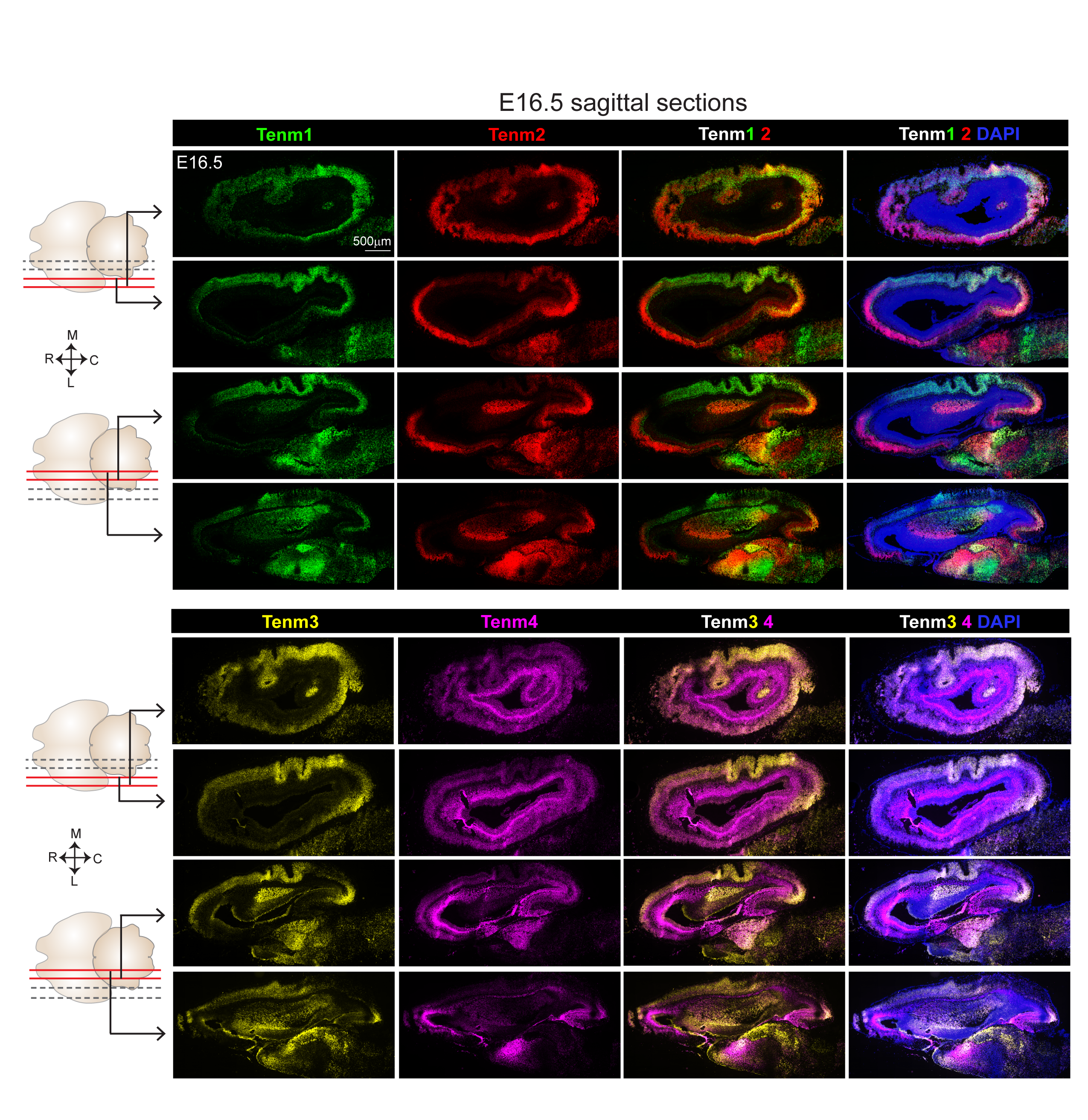

Supplement: S2 Fig — Location of each sagittal section in lateral-medial axis is shown through the cartoons on the left side. Images in this figure are re-used in Fig 2A. (TIF) [file pbio.3002599.s002.tif]

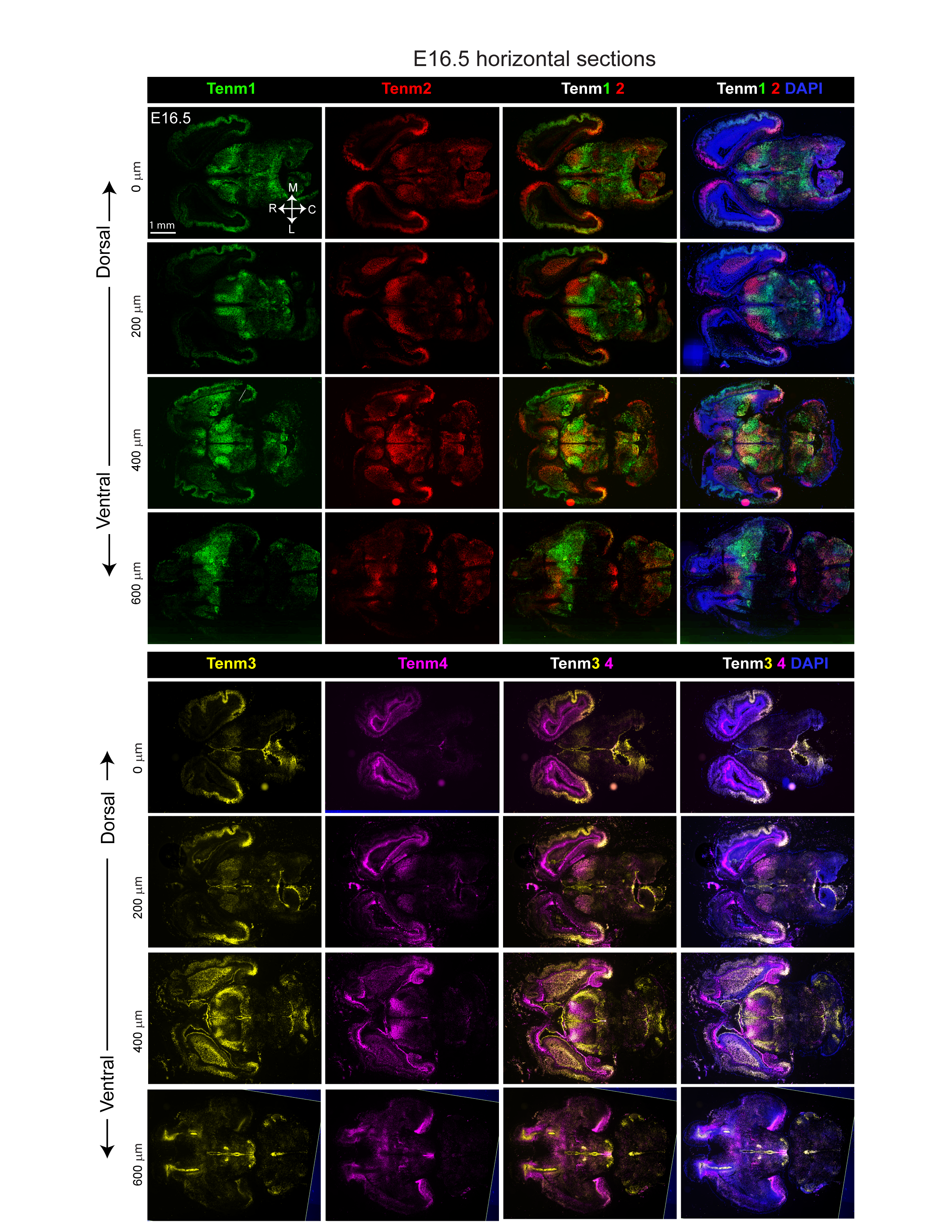

Supplement: S3 Fig — (TIF) [file pbio.3002599.s003.tif]

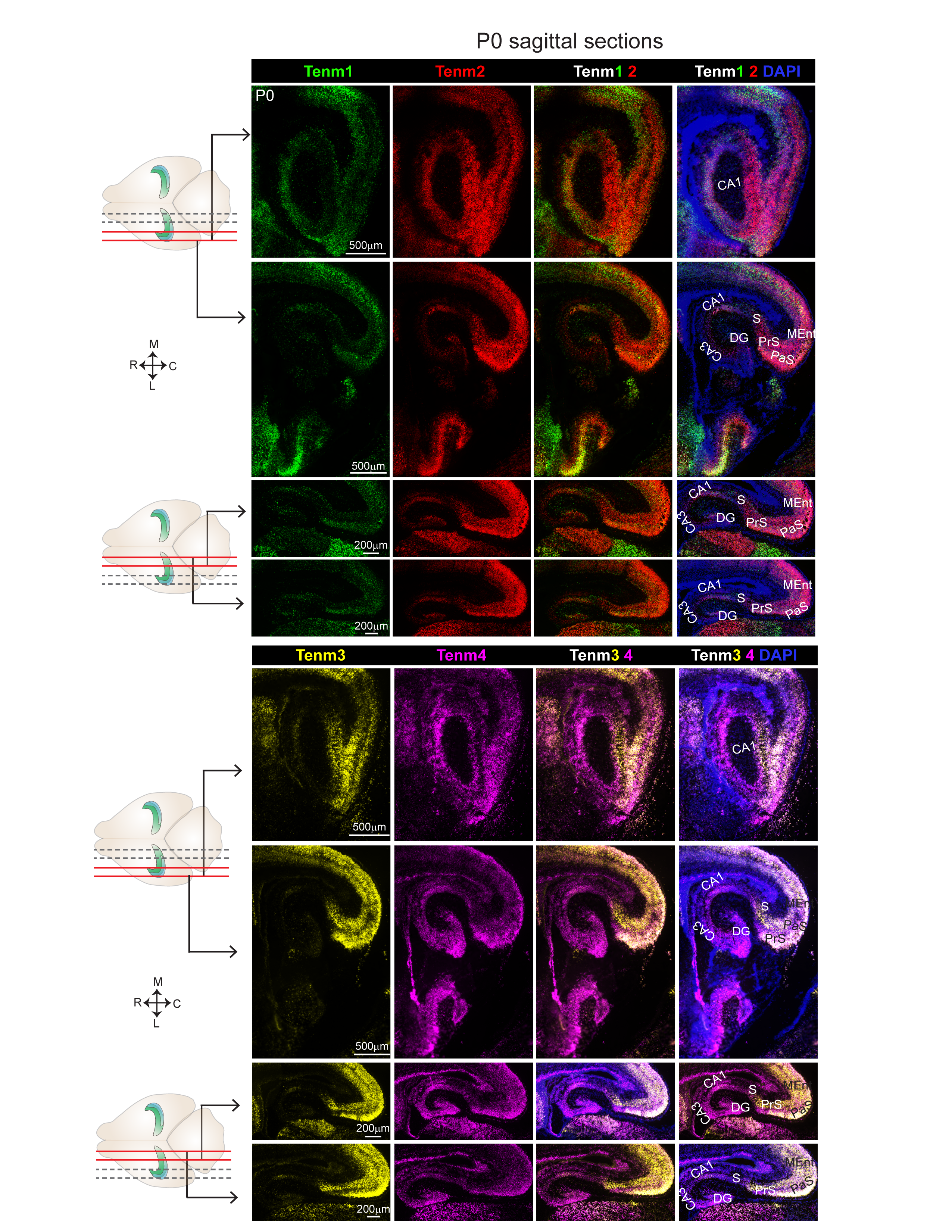

Supplement: S4 Fig — Location of each sagittal section in lateral-medial axis is shown through the cartoons on the left side. Images in this figure are re-used in Fig 2B. S–subiculum; PrS–presubiculum; PaS–parasubiculum; CA1, CA3 –cornu Ammonis 1, 3; DG–dentate gyrus; MEnt–medial entorhinal cortex. (TIF) [file pbio.3002599.s004.tif]

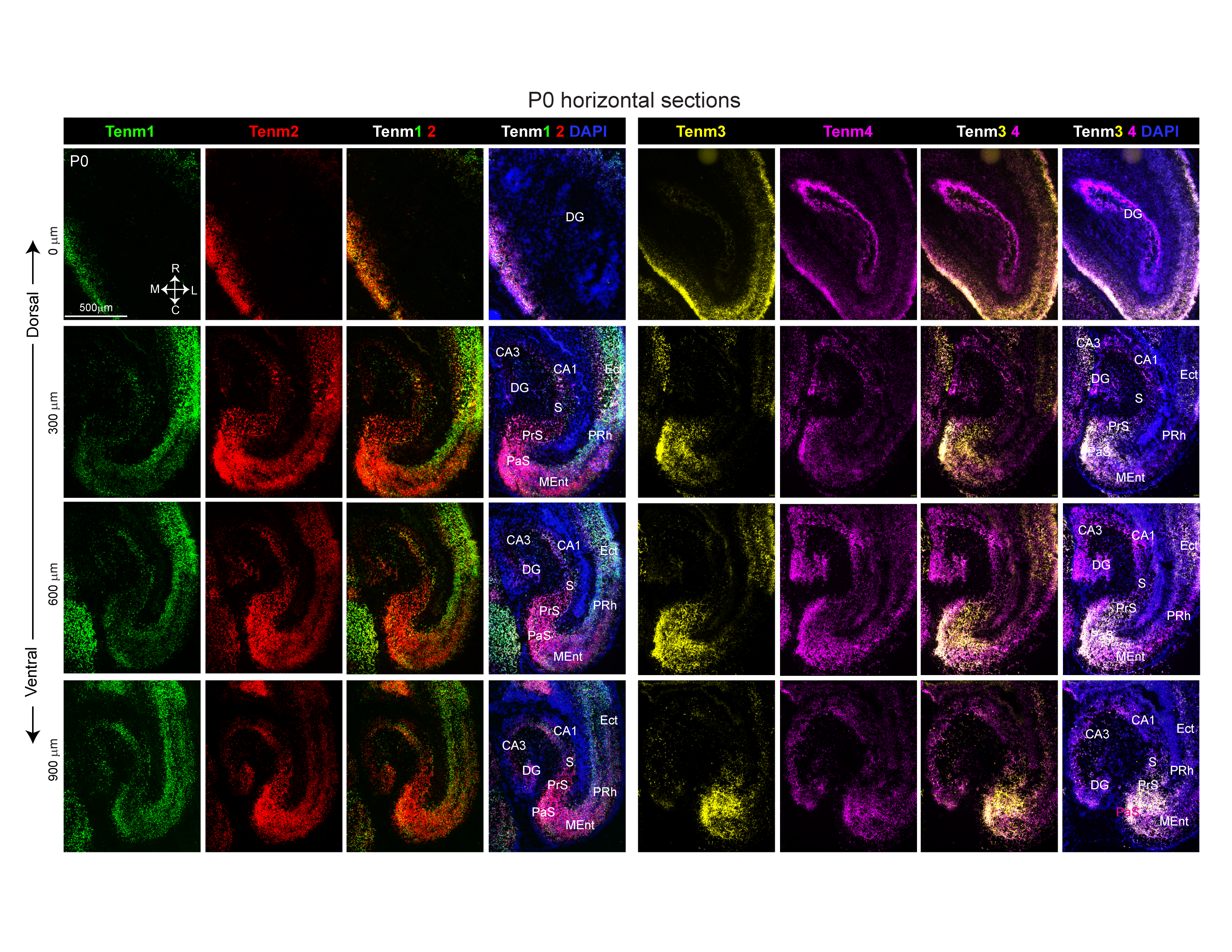

Supplement: S5 Fig — S–subiculum; PrS–presubiculum; PaS–parasubiculum; CA1, CA3 –cornu Ammonis 1, 3; DG–dentate gyrus; MEnt–medial entorhinal cortex; Ect–ectorhinal area; PRh–perirhinal area. (TIF) [file pbio.3002599.s005.tif]

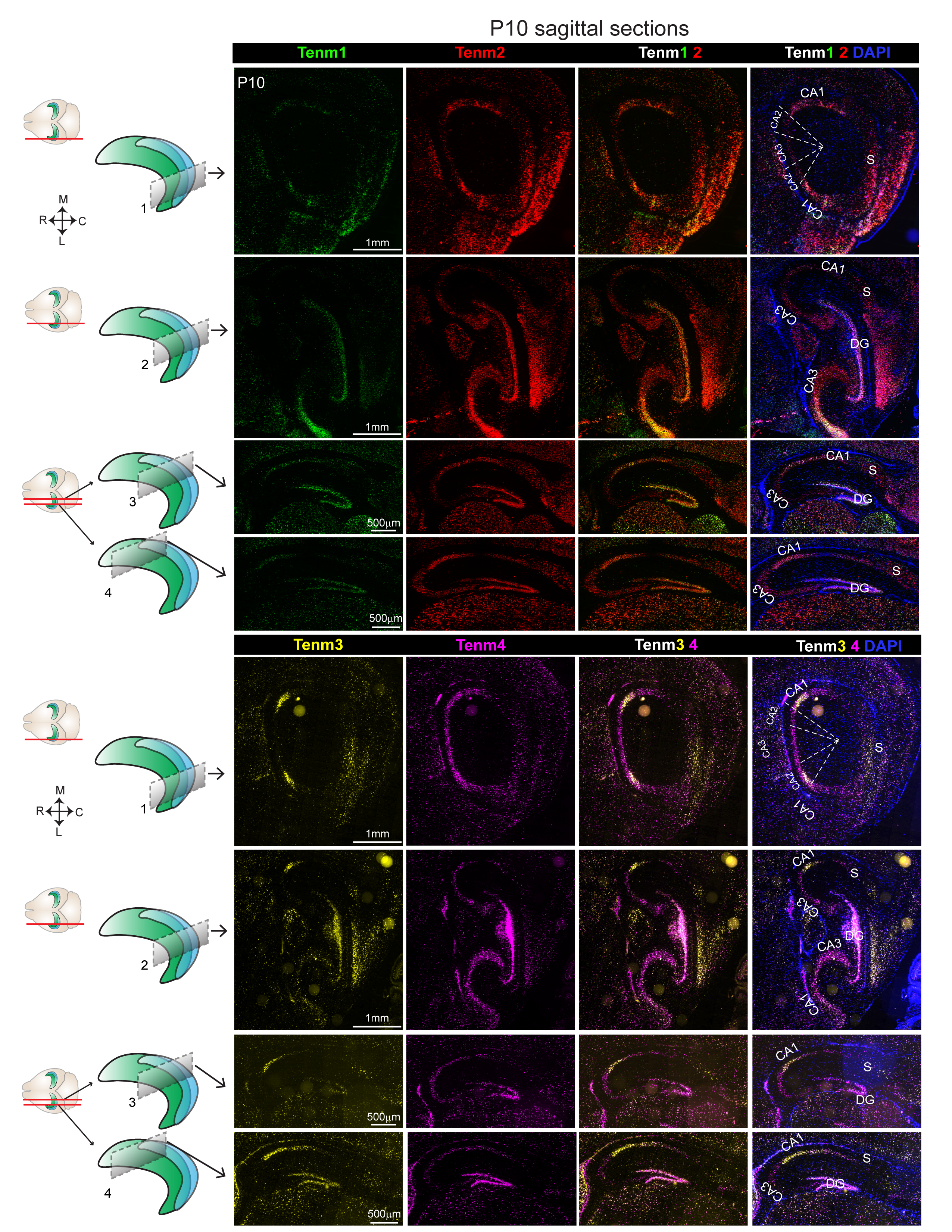

Supplement: S6 Fig — Location of each hippocampal sagittal section in lateral-medial axis is shown through the cartoons on the left side. Images in this figure are re-used in Fig 2E. S–subiculum; CA1, CA2, CA3 –cornu Ammonis 1, 2, 3; DG–dentate gyrus. (TIF) [file pbio.3002599.s006.tif]

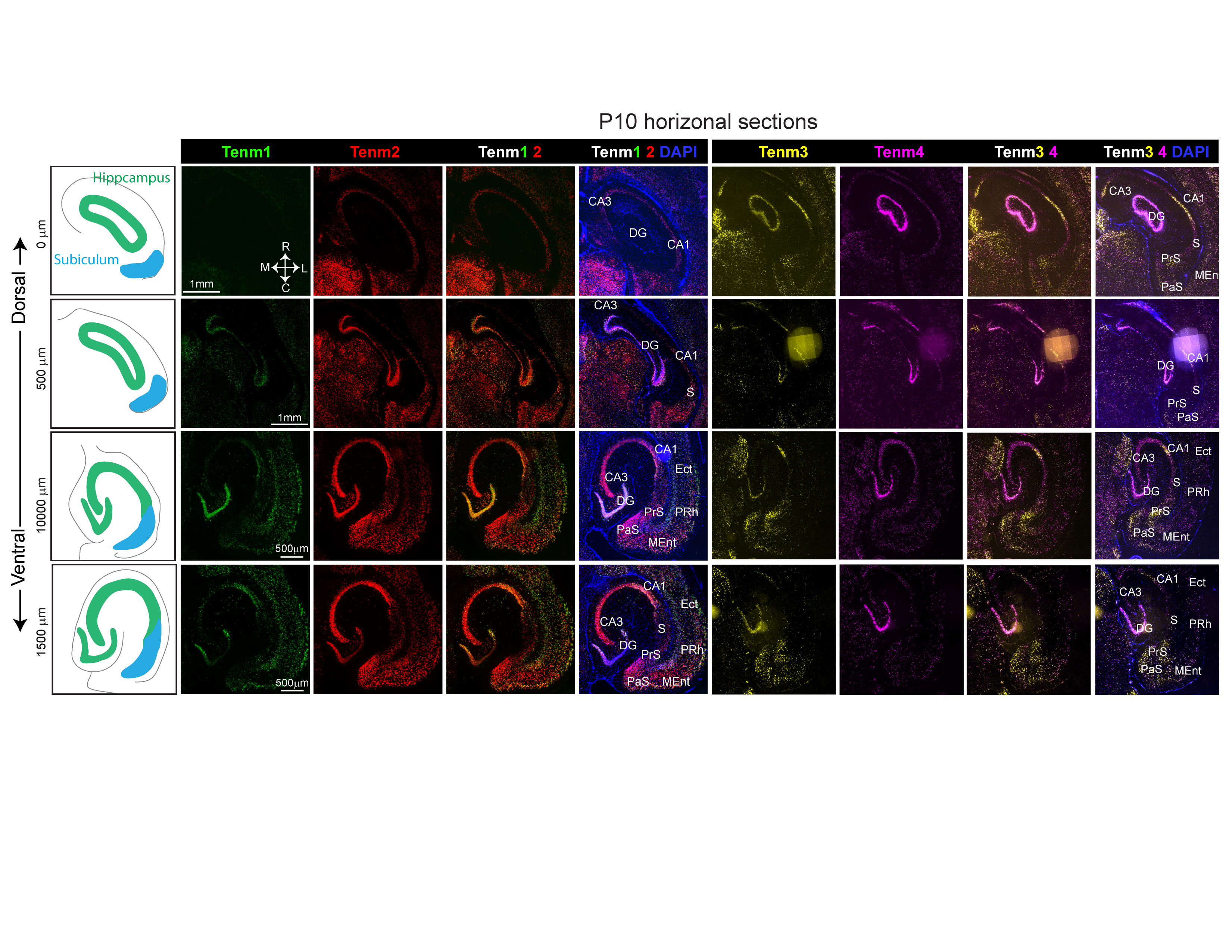

Supplement: S7 Fig — Location of each hippocampal horizontal section in dorsoventral axis is shown through the cartoons on the left side. Images in this figure are re-used in Fig 7E. S–subiculum; PrS–presubiculum; PaS–parasubiculum; CA1, CA3 –cornu Ammonis 1, 3; DG–dentate gyrus; MEnt–medial entorhinal cortex; Ect–ectorhinal area; PRh–perirhinal area. (TIF) [file pbio.3002599.s007.tif]

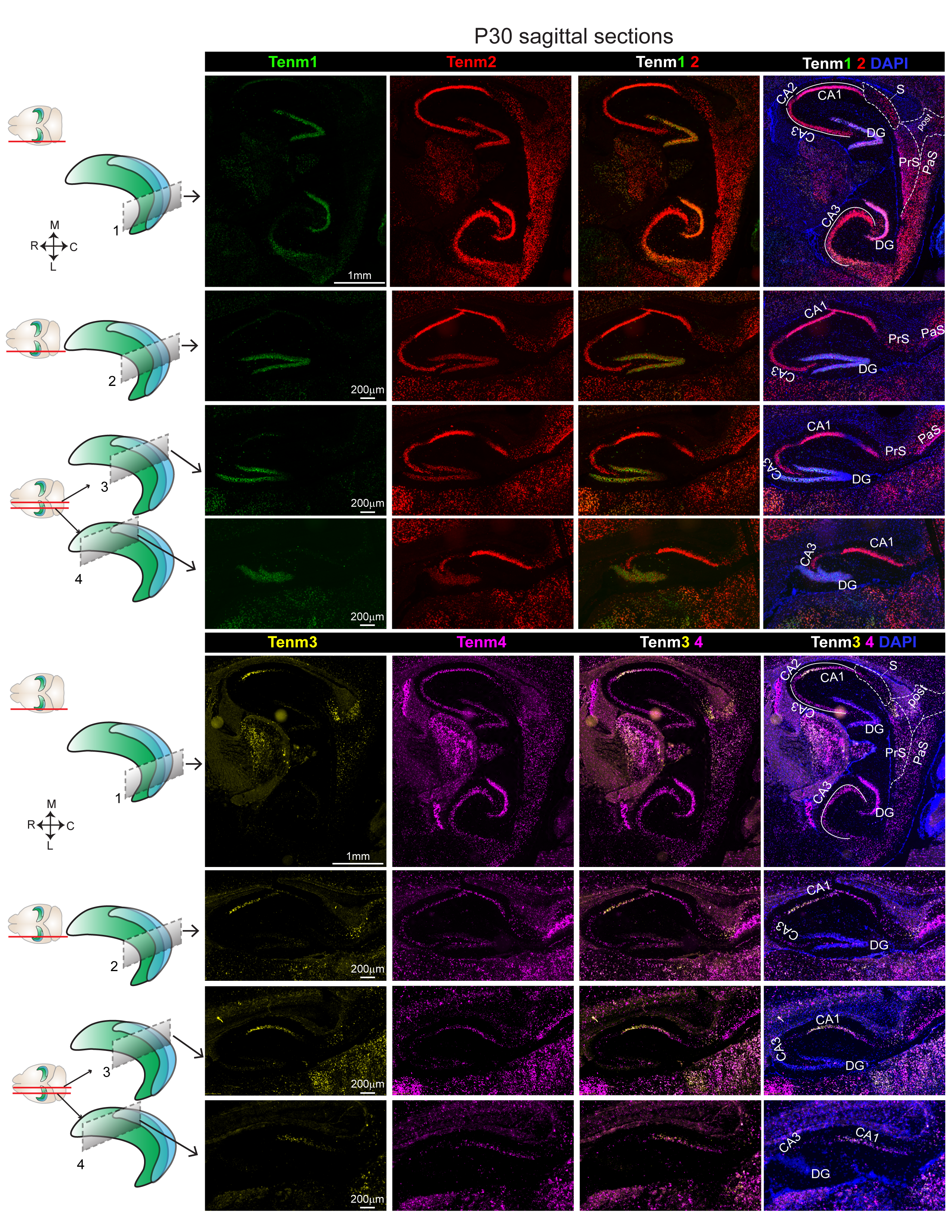

Supplement: S8 Fig — Location of each hippocampal sagittal section in lateral-medial axis is shown through the cartoons on the left side. Images in this figure are re-used in Fig 2F. S–subiculum; PrS,–presubiculum; PaS–parasubiculum; Post–postsubiculum; CA1, CA2, CA3 –cornu Ammonis 1, 3; DG–dentate gyrus; MEnt–medial entorhinal cortex; Ect–ectorhinal area; PRh–perirhinal area. (TIF) [file pbio.3002599.s008.tif]

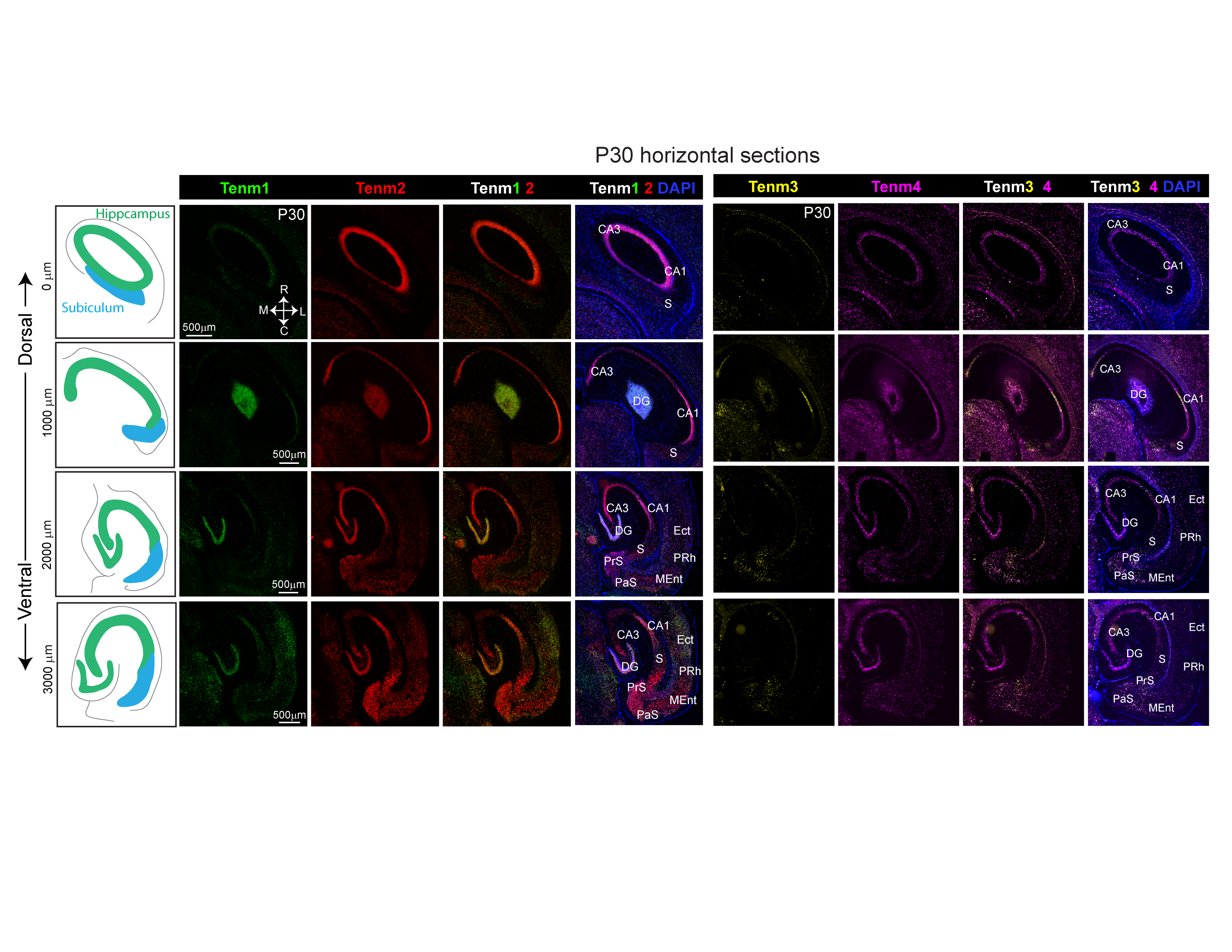

Supplement: S9 Fig — Location of each hippocampal horizontal section in dorsoventral axis is shown through the cartoons on the left side. Images in this figure are re-used in Fig 7E. Sub–subiculum; PrS,–presubiculum; PaS–parasubiculum; CA1, CA3 –cornu Ammonis 1, 3; DG–dentate gyrus; MEnt–medial entorhinal cortex; Ect–ectorhinal area; PRh–perirhinal area. (TIF) [file pbio.3002599.s009.tif]

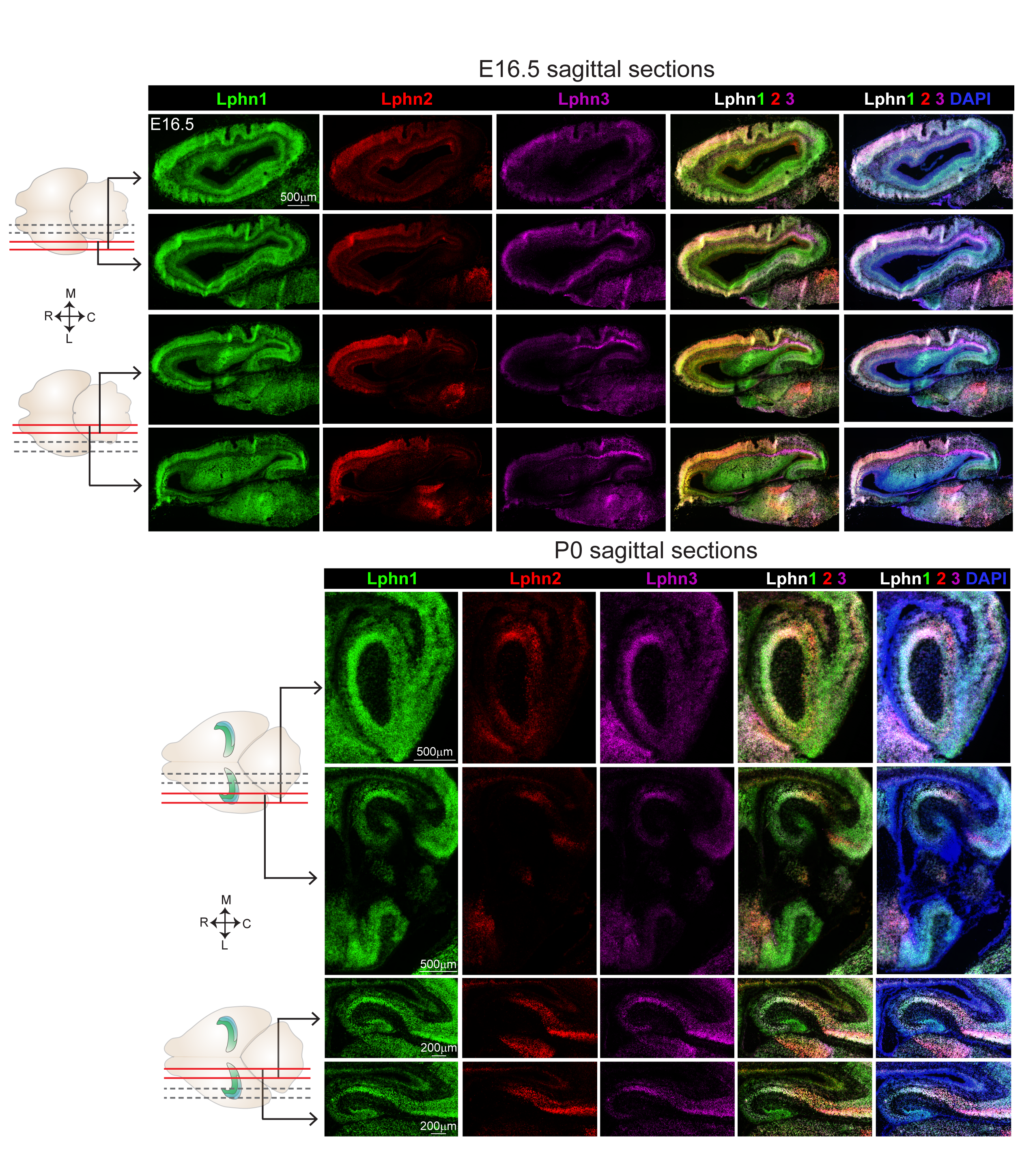

Supplement: S10 Fig — Microscopic images of sagittal sections of hippocampal region from E16.5 (top panel) and P0 (bottom panel) mouse brains labeled with RNA in situ hybridization probes to detect Lphn1, Lphn2, and Lphn3 expression. Location of each sagittal section in lateral-medial axis is shown through the cartoons on the left side. Images in this figure are re-used in Figs 4A, 4B and 5D. (TIF) [file pbio.3002599.s010.tif]

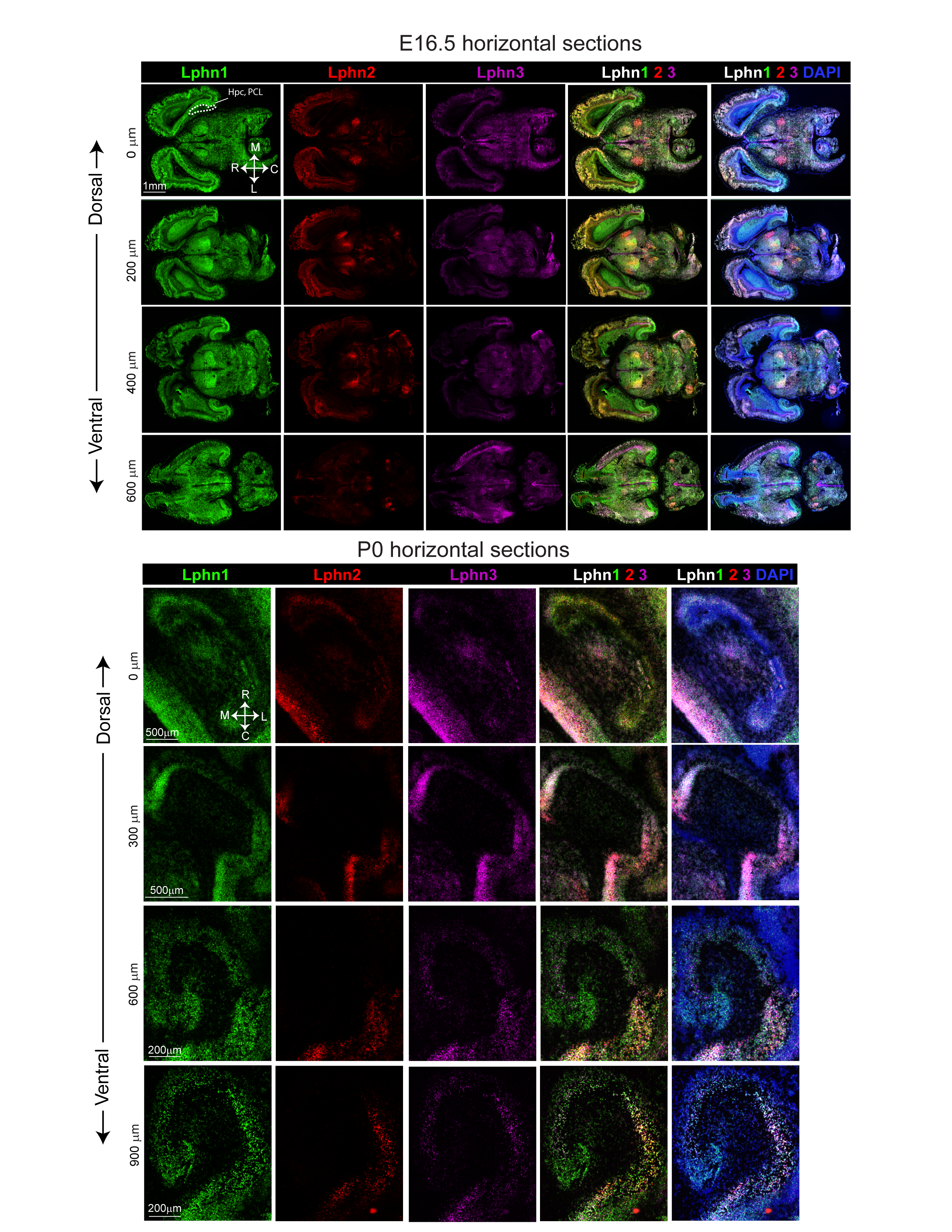

Supplement: S11 Fig — Microscopic images of horizontal sections of hippocampal region from E16.5 (top panel) and P0 (bottom panel) mouse brains labeled with RNA in situ hybridization probes to detect Lphn1, Lphn2, and Lphn3 expression. (TIF) [file pbio.3002599.s011.tif]

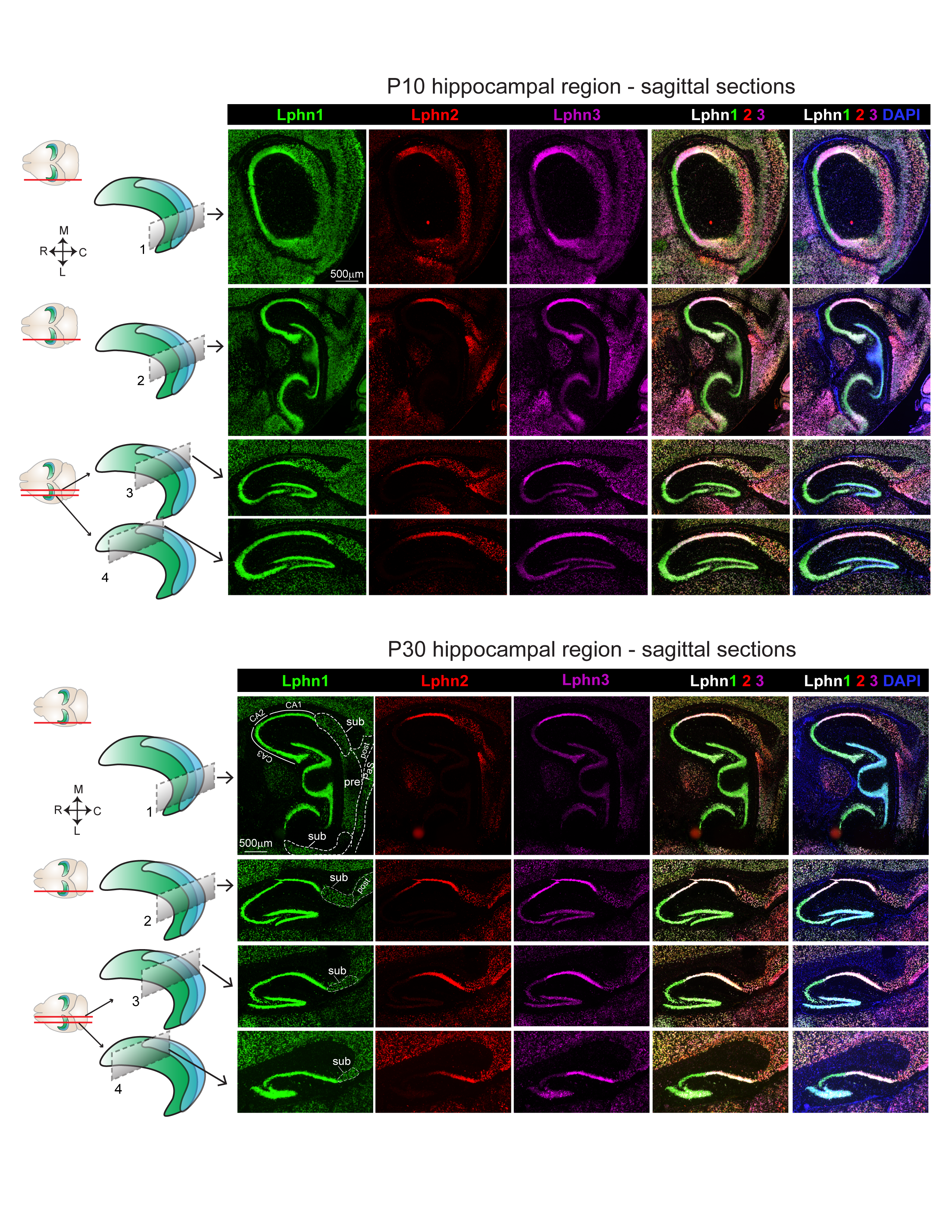

Supplement: S12 Fig — Microscopic images of sagittal sections of hippocampal region from P10 (top panel) and P30 (bottom panel) mouse brains labeled with RNA in situ hybridization probes to detect Lphn1, Lphn2, and Lphn3 expression. Location of each hippocampal sagittal section in lateral-medial axis is shown through the cartoons on the left side. Images in this figure are re-used in Figs 4E, 4F and 5E. S–subiculum; PrS–presubiculum; PaS–parasubiculum; post–postsubiculum; CA1, CA3 –cornu Ammonis 1, 3; DG–dentate gyrus; MEnt–medial entorhinal cortex; Ect–ectorhinal area; PRh–perirhinal area. (TIF) [file pbio.3002599.s012.tif]

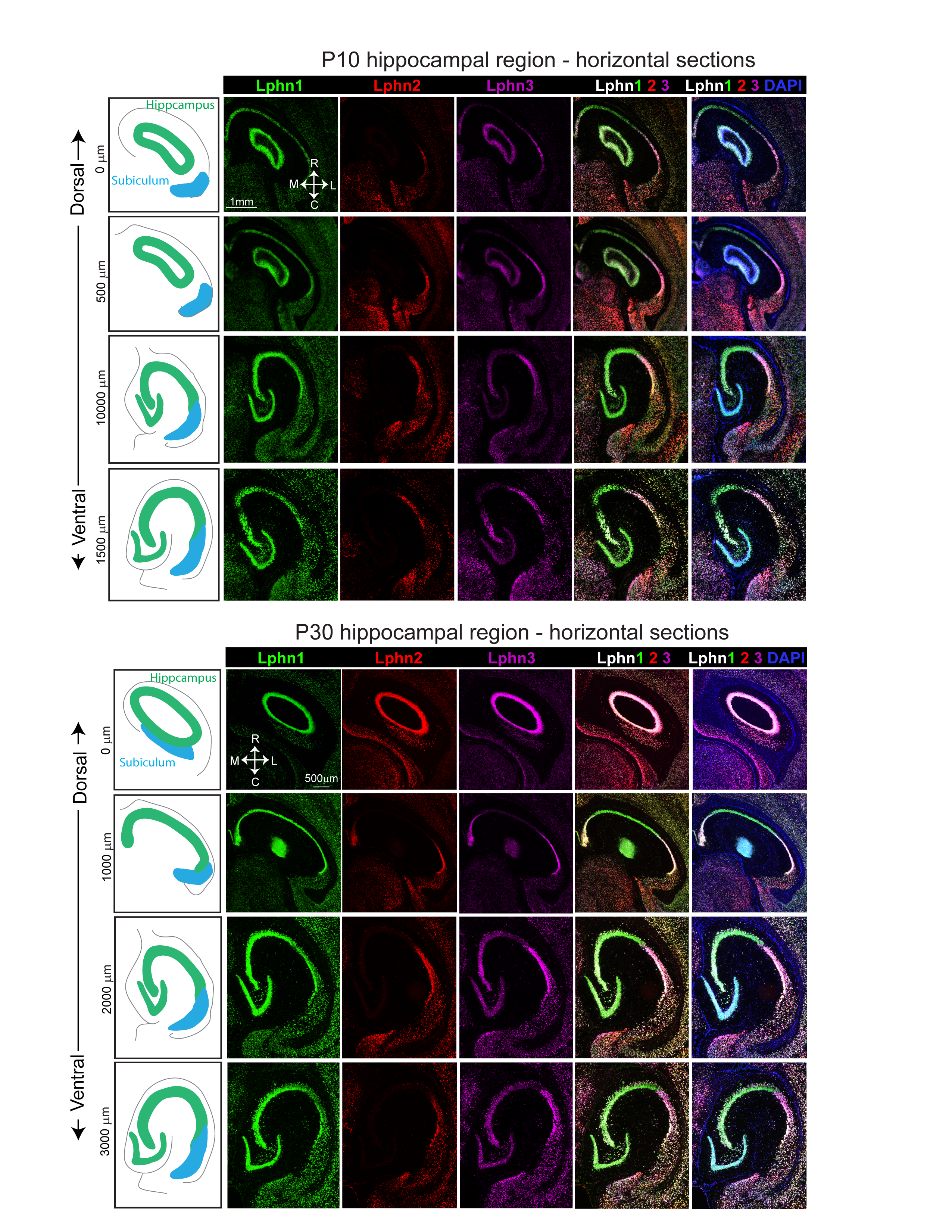

Supplement: S13 Fig — Microscopic images of horizontal sections of hippocampal region from P10 (top panel) and P30 (bottom panel) mouse brains labeled with RNA in situ hybridization probes to detect Lphn1, Lphn2, and Lphn3 expression. Location of each hippocampal horizontal section in dorsoventral axis is shown through the cartoons on the left side. Images in this figure are re-used in Figs 5E, 6A and 6B. (TIF) [file pbio.3002599.s013.tif]

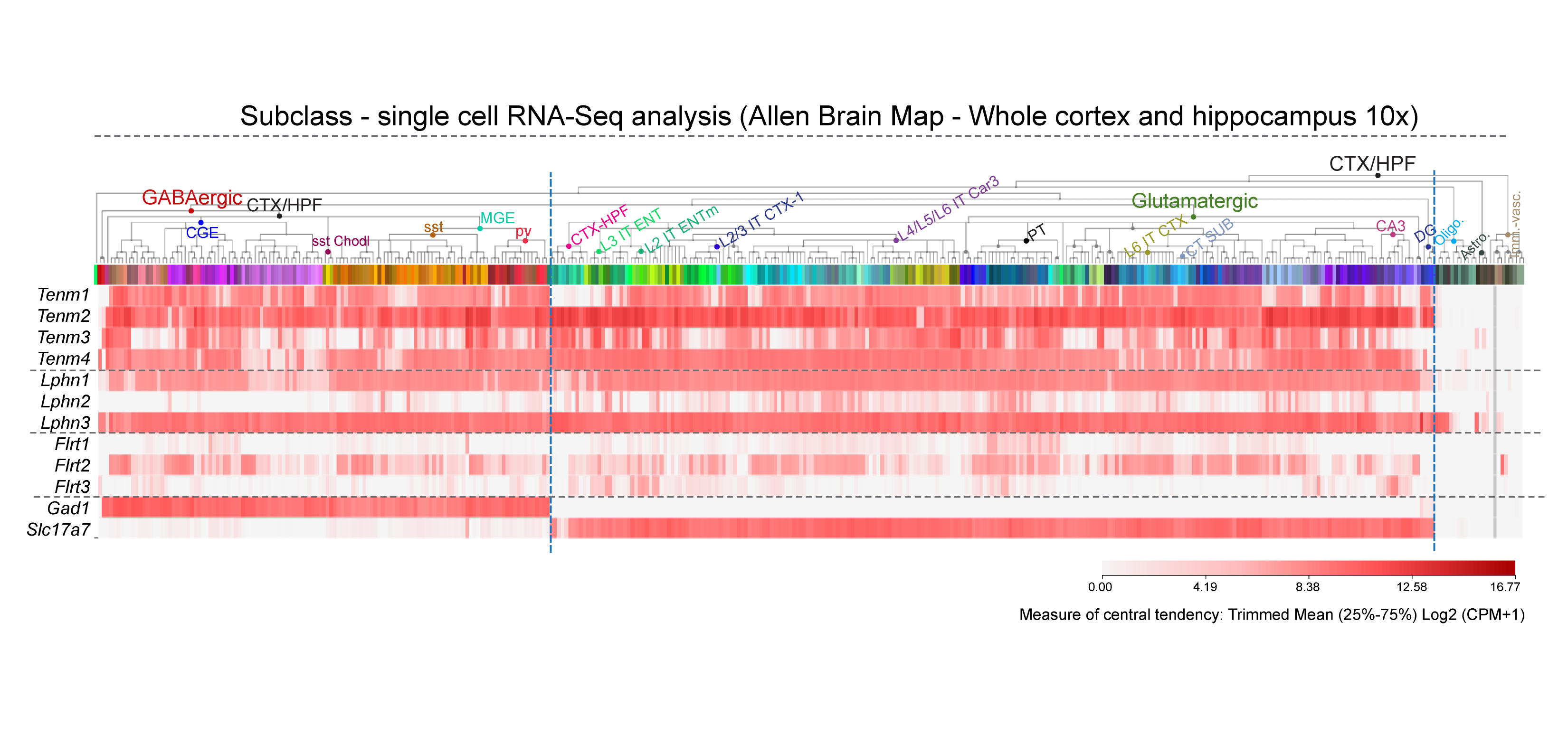

Supplement: S14 Fig — GABAergic cell marker Gad1 and Glutamatergic cell maker Slc17a7 (Vglut) expression levels are also shown in the bottom for quality control. Whole cortex and hippocampus scRNA-seq data obtained from Allen Brain Map - https://celltypes.brain-map.org/rnaseq/mouse_ctx-hpf_10x. Various cell types of GABAergic and glutamatergic neurons are indicated in different colors. (TIF) [file pbio.3002599.s014.tif]

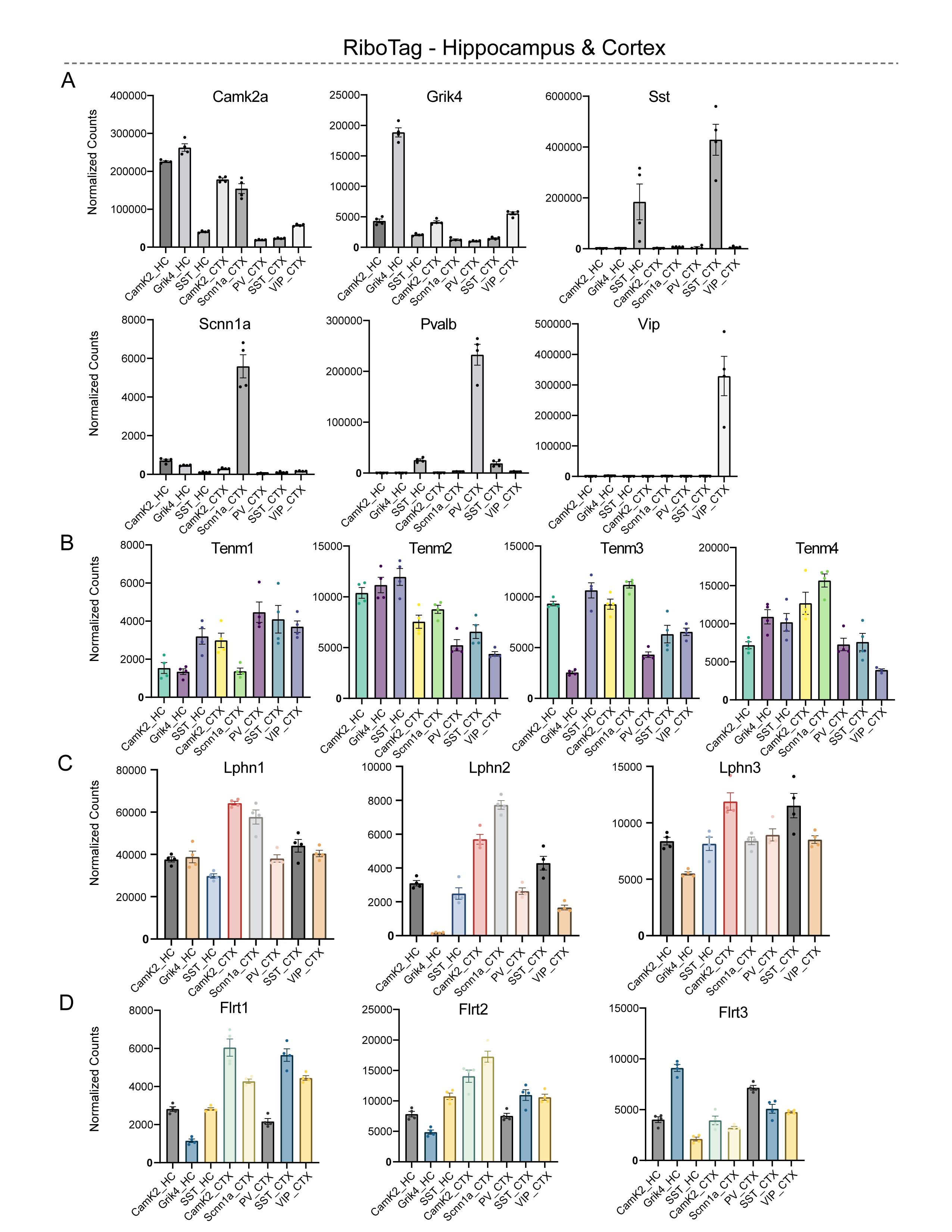

Supplement: S15 Fig — (A) Bar graphs show expression levels of excitatory (Camk2a and Grik) and inhibitory (Sst, Scnn1a, Pvalb, and Vip) cell markers (as quality control) in respective cell populations as revealed by sequencing of ribosome-engaged total mRNA (RiboTag seq). Bar graphs show expression levels of Tenms (B), Lphns (C), and Flrts (D) in RiboTag inhibitory and excitatory cell populations in the hippocampus (HC) and cortex (CTX). RNA-seq data obtained from Splicecode database (https://scheiffele-splice.scicore.unibas.ch/). The data underlying graphs in A–D can be found in S1 Data. (TIF) [file pbio.3002599.s015.tif]

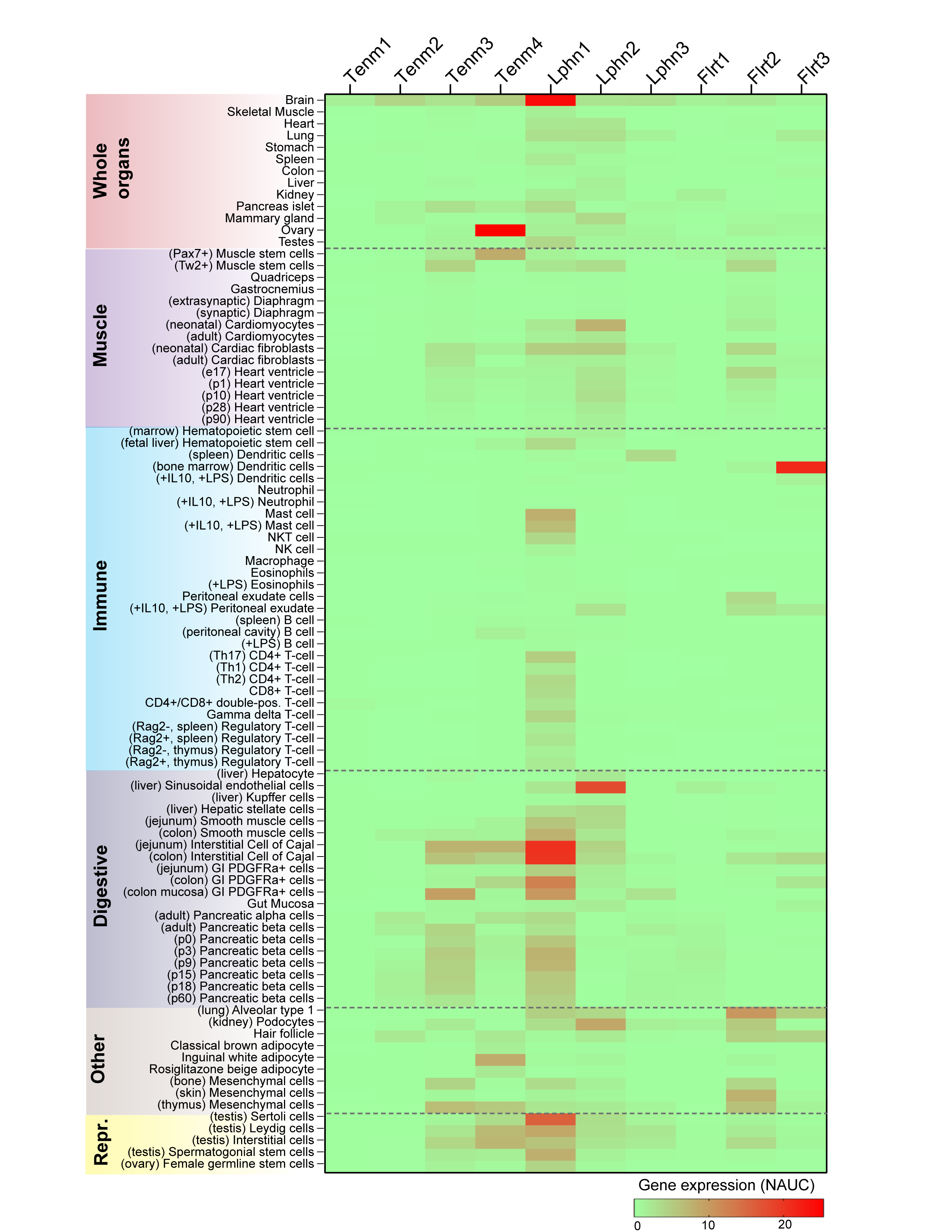

Supplement: S16 Fig — Note the high expression of Lphn1 in several non-neural tissues. RNA-seq data obtained from ASCOT database (http://ascot.cs.jhu.edu/). The data underlying this figure can be found in S1 Data. NAUC–Normalized Area Under the Curve. (TIF) [file pbio.3002599.s016.tif]
