# Supplementary material for: Cartography of teneurin and latrophilin expression reveals spatiotemporal axis heterogeneity in the mouse hippocampus during development
Source: PLoS Biol. 2024 May 7;22(5):e3002599. doi: 10.1371/journal.pbio.3002599 (PMC11101112; doi:10.1371/journal.pbio.3002599)
Supplement: Attachment — Submitted filename: Reviewer comments_response.docx [file pbio.3002599.s018.docx]

Reviewer #1: Liakath-Ali et al present a well-written paper with beautiful figures that presents the spatial and temporal distribution of synaptic adhesion molecules, two of which have been shown to be involved in embryonic hippocampus development. The validation of their in situ hybridization using RNAseq databases is clever and provides a nice example of the utility of these databases in studies like this. That being said, this article is not clearly presented as a methods paper, and, as such, my comments are about framing their results in the broader picture of hippocampus development because that is how they have begun to frame the work. I think that if the intention is for this paper to be a methods paper, the authors should reframe their work in that regard.

We thank the reviewer for recognizing our efforts in this study and providing constructive feedback.

I have a few comments that I think would increase the strength of the paper.

1)  In the abstract the authors present clear logic as to why the focus of the paper is on tenuerins and latrophilins (because of the role of Tenm3 and Lphn2 in embryonic hippocampus development), and this should be carried into the Introduction. Adding this information to the introduction would make the reasoning behind the study more robust and cohesive.

We added this now in the Introduction section (Page 3).

The authors also note that these SAMs are evolutionarily conserved during development? It would be interesting to comment if these are the only SAMs that are evolutionarily conserved, and also to note that connectivity of the hippocampal-parahippocampal regions is itself heavily conserved across species. This is likely a point for the discussion, but it might help highlight the relevance and role of these SAMs in the hippocampal-parahippocampal development.  

Many SAMs are evolutionarily conserved and we agree that this expression could have contributed to the construction of hippocampal-parahippocampal connectivity. We added this in the discussion section (Page 13).

2)    The description of the development of the hippocampus in the introduction could be clarified and should include references. The description that neurogenesis primarily occurs in the dentate gyrus and subventricular zone is something that is described a lot for adult neurogenesis, and is even described for adult neurogenesis in the reference provided by the author (19). However, because the dentate gyrus is a later emerging structure, it is unclear why the authors describe this in the adult terms. For example, many developmental biologists would instead say that origin of hippocampus neurons (neurogenesis) in the developing embryo includes glutamatergic cells produced locally by progenitors in the ventricular zone of the primordial hippocampal area (Cossart and Khazipov, Physiological Reviews 2022). Interneurons arise from the median ganglionic eminence. Additonally, I understand that the current study is performed in mice, but in the rat, about 90 percent of the dentate granule cells are born after birth of the pup, therefore, the current language used to describe neurogenesis in the hippocampus seems misleading, and I personally think it would be better to phrase the origin of hippocampus neurons in the more common developmental terms.
We now reframed and expanded this part in the introduction with additional references. (Page 3, 4)

3)    The authors introduce very broadly the role of the dorsal and ventral hippocampal poles with very few references on the topic. For example, the few references that the authors include are not the primary references for the connectivity of the hippocampus with the structures listed in the introduction. It would be good to read and include some primary references.

We now added three primary references (Swanson, Cowan 1977; Moser et al 1995 and Henke 1990) that originally showed the distinct input and output connections in ventral and dorsal hippocampus, dependence of spatial memory on the dorsal hippocampus and emotional behavior and affective processes on the ventral hippocampus. (Page 4).

Methods
1)    The authors do not indicate how the timed breedings were performed to collect E16.5 embryos, or how postnatal day 0 was determined. If timed breeders were ordered from Charles River, the authors should confirm that they have the gestational days they expected. My experience with Charles River is that they call the day the plug is observed day 1 (instead of the widely accepted 0.5) and therefore, timed breeders purchased from Charles River are actually a day earlier in the gestational period.

We did not order timed breeders from Charles River. However, we used WT mice from Charles River to set up in-house timed mating to obtain E16.5 embryonic mice using standard vaginal plug identification method. This information is now added in the methods section. (Page 15, 16)

2)    It is confusing and unclear what the authors are referring to when they say that "four lateral-medial (sagittal) and dorsal-ventral (horizontal) cryosections with an interval of 0.2 mM (for E16.5), 0.5 mM (P0), 0.75 mM (P10) and 1.0 mM (P30) were collected. mM what? Is this in reference to the concentration of the solution that the slices were incubated in? Is there a typo in the units such that it should be mm?
We apologize for this typo. They were meant to be millimeter (mm) interval for obtaining brain sections. This is now corrected. (Page 16).

Results

Figure 2
The authors should indicate on the embryonic tissue what they are delineating as each hippocampal region. It would be useful for the authors to include a schematic of the developing brain at E16.5 with the CA1, CA2, and CA3 regions identified. It also seems that from the DAPI stain, the authors should be able to delineate the layers of CA1-CA3 in the P0 brain, though this is hard to see with the SAMs overlaid as well.
For our prenatal and neonatal stage hippocampal signal quantifications, we relied on the Mouse Brain Atlas manual by Uta Schambra (2008 edition). This is to ensure that we follow well-curated anatomical annotations of hippocampal regions. Unlike the detailed Allen Mouse Brain Common Coordinate Framework that provide high resolution annotations, this manual doesn’t delineate CA1-CA3 regions. We however, asked if we remove the brain from the skull to slightly unfold and straighten would help in identifying the regions with their layers (usually the embryonic brain are cut as intact with the skull, where its original posture is retained). Due to the crowded nature of cells, as seen using DAPI, we could not identify CA regions with their layers. And, the removal of embryonic brain out of the skull and embedding within the cryosectioning solution further flattened the brain and altered its original posture. Hence the hippocampus appears as an anterior structure in our images. We now included this explanation in the method section (Page 14, lines 14-17). We believe that this information and the schematic provided as an inset in the figure would help readers to see the results clearly.

It is also unfortunate that the color scheme representing the layers of the hippocampus has a high level of overlap with the colors used to label the tenuerins in the ISH slides. The authors should consider changing the color scheme of the hippocampus layers.
We agree that the color scheme of the bubble plots are confusing. We now changed the original color of the bubbles to darker in all the plots presented in the Figure 2 and 4.

Figure 3
I think the authors did a nice job of representing the data in panels A through D. I like the representation of the mean intensity and how they go about translating the regions from age to age. This is easy to follow. Challenging to follow is the lateral to medial staining (until I got to Figure 5, where the lateral to medial sections are easier to identify and follow). In Figure 3, the medial and lateral sections do not appear very different, whereas the authors present the full range of lateral to medial sections used in Figure 5. A schematic of the laterality represented in each pane for panel E would be helpful (schematic of lateral to medial sections).

We are glad that the spatiotemporal heatmap panels are useful to follow the results. In panel E we did not intend to show any temporal expression information of Tenm1-4. The two sections in P10 and P30 are separate images that show Tenm1, Tenm2 and, Tenm3 and Tenm4 separately. We showed these results to highlight the striking reduction of Tenm3 in P30 s.p. layer (CA1) and in all entorhinal layers. We also show Tenm4’s reduction in all layers of entorhinal cortex in P30 when compared to P10. We updated this information in Figure 3 caption. To illustrate the overall orientation of different sections, we have provided a detailed schematic in S. Fig 1B. In addition to this, wherever possible we provided schematic of sections throughout supplementary figures that contain in-situ images.

Figure 6
It is unclear to me why the figure title refers to the expression is as a gradient. The staining in the respective layers looks consistent along the dorsal-ventral axis.
This was a typo. This is now corrected as “Latrophilin expression is relatively constant across hippocampal dorsoventral axis”.

When describing the data in the results for this figure, the authors state that "With horizontal sections, we were able to capture two dorsoventral planes of EC." It is really unclear what they mean by this. What are the two dorsoventral planes?
We meant that the EC regions were identified from two dorsoventral sections. This sentence is now corrected to “With horizontal sections, we were able to identify EC regions from two dorsoventral -sections” (Page 8).

That authors go on to say that "In P30, the ventral-most EC (caudomedial entorhinal cortex) regions showed striking enrichment of Lphns in multiple layers with high levels of Lphn1 in L2 and almost no expression of all Lphns in L1." Thinking forward about the discussion, the authors should indicate where in the cell these proteins are found. Layer 1 is a relatively cell-free layer that consists mostly of fibers, so it isn't surprising to not see label here when all the other RNA label appears to be seen in cell bodies.
If we understood this comment correctly, Lphn mRNA primarily found in cell bodies. We do see a sparse presence of cells in L1 as seen through DAPI but most of these cells don’t have Lphn expression (see merged image below).

Discussion
1)    On page 12, lines 6-8, the authors should provide a reference for the statement that the selective expression of Tenm, Lphn and Flrt paralogs outside of the CNS suggests that they may play similar cell-adhesion roles in relevant tissues.

We now added reference for this section (Page 13).

2)    On page 13, lines 5-7, the authors should provide a reference for the critical period for experience-dependent plasticity in CA1, and they should additionally state what this time period is. The authors find that Tenm1 peaks at P10 and likely the authors mean that this coincides with the critical period for experience-dependent plasticity, but they also should recognize that the window of P10 to P30 is quite a lot of time when it comes to development and stating the critical window would help put their finding into perspective.
We understand that the critical period for experience dependent plasticity in sensory cortex (P10-20) is well known. Upon a literature search and personal communication with experts in the area, we realized that critical period for CA1 is rather elusive. However, there are some studies pointing to specific time window for cellular plasticity (eg. Tashiro et al J. Neurosci., 2007; Bergami et al., Neuron 2015; Carstens et al., J. Neurosci., 2016; Khoo et al., Mol Neurobiol). For CA1-specific experience-dependent critical period (mainly response to stressors), one study points possible time window between P17 and P24, in rats. We slightly modified our statement in light of these references. (Page 14).

3)    The terminology using "mediolateral" is confusing, especially because the authors try to translate it to dorsal ventral using the HippoSeq data (Figure 7). Why not simply delineate the dorsal and ventral portions of the hippocampus from the sagittal sections to begin with? This would also help with the nomenclature that is used by others with respect to medial and lateral being based on looking at sections from the horizontal or coronal orientations. It seems that the authors should have the ability to delineate the dorsal and ventral portions of the hippocampus from the sagittal views based on the Supplementary Figures 1, 6 and 8. In taking the average intensity of the sections base on the laterality, there certainly would be an average of dorsal and ventral portions of the hippocampus in some sections that could hide increased intensity in either pole. If the point is to be able to compare across ages, the authors should consider doing both the dorsal-ventral delineations and the laterality, recognizing that in the case of the laterality, the embryonic hippocampus doesn't have the same amount of curvature and therefore the direct comparisons are difficult to make.

This is Figure 8 now. The whole datasets with anatomical registration and quantification in this study was performed on sagittal sections covering mediolateral regions, mainly because of the availability of reference annotation from Allen atlas and hard copy manual for prenatal and postnatal brain. For dorsal ventral quantifications, the horizontal datasets (presented in S. Fig 3, 5, 7, 9, 11 and 13) could’ve been useful. However, there is no high-resolution annotated brain anatomy is available for horizontal sections for various developmental stages. This curbed our ability to use our algorithm for dorsal ventral quantification. Nevertheless, we provided all the horizontal image datasets in the supplementary figures and would be more than happy to provide high-resolution raw images to the community for further quantifications, upon request. The mediolateral nomenclature and quantification is also widely used in various studies (For eg. Roth et al 2015, Nat Neurosci; Munoz-Castaneda et al 2021, Nature; Allen et al 2016, J Physiol; Buhusi et al 2008, J. Neurosci).

Importantly, the authors could include more information in the methods to describe why they decided to take measurements and average the way they did.

We have now added this information in the methods section.

4)    Finally, the authors could do more to place their results into the larger framework of hippocampus development and function. As written, there is information about expression of genes in the dentate gyrus and CA1 that may relate to function in these regions, but the authors do not say much in regard to the role of the SAMs in these processes. Furthermore, the authors suggest that the reader could use the data to generate hypotheses, but it seems like the authors could also suggest some hypotheses, as they are experts in SAMs, in order to help place their results into perspective.
We have added examples on how the hypothesis on the functional dichotomy of spatial expression can be tested (Page 13).
Minor points:
1)    There are a number of abbreviations that should be spelled out (e.g. FPKM in the y-axis of Figure 7, NAUC in Figure 8) for the reader with little RNAseq experience.
We have now added these abbreviations in Fig. 7 and 8 captions.

2)    The use of deep and superficial in the Supplementary Figure 2 is also confusing. Deep and superficial typically are used for terminology of looking at the cells in the CA layers. Why not call it dorsal and ventral here?
We agree. This is now changed in Supplementary Figure 3, 5, 7, 9, 11 and 13.

3)    Again, in the supplemental figures it would be nice to see a schematic of what was used to delineate the hippocampal regions in the embryonic hippocampus, in particular in the very lateral sections.
Pls see comments above in response to comment on Figure 2.

Reviewer #2: The new manuscript provides explicit spatiotemporal information on how teneurins and latrophilins are expressed in the brain during development and after birth. Since these molecules (as cell adhesion molecules) are involved in forming a transsynaptic complex - such information can be helpful for those neuroscientists who study synapse formation and maintenance. All experiments are qualitatively done, and I have no complaints about the research protocol and result interpretation.
However, my main concern is that the study is purely descriptive (which is not always bad), based on a relatively standard methodology of labeling the group of seven specific targets. As a result, the current view of the problem was supplemented by some details. In addition, the authors used previously existing resources (RNAseq) but did not create them. So I can not classify the new resource (despite the many advantages of the study) as "exceptional and spurring future research." However, in my opinion, this study can be published as it is in journals that are focused on the comparative and developmental aspects of neuroscience.

We appreciate this reviewer’s comments on our manuscript and are grateful for the recognition of the potential impact our findings may have on the neuroscience community. We understand the concern regarding the descriptive nature of our study and the utilization of previously existing resources, but we believe there are several compelling reasons to consider the significance of our work. Firstly, while the methodology we employed may be considered standard in some respects, the comprehensive spatiotemporal information we have provided on the expression patterns of teneurins and latrophilins during brain development and postnatal stages fills a critical gap in the field. Our qualitative approach offers detailed insights into their expression dynamics, which can serve as a foundational reference for further investigations into synaptic development and function. While we did not generate new RNA-seq data in this study, we meticulously validated our observations using existing datasets. Leveraging these resources not only ensures the reproducibility of our findings but also enhances the robustness of our analyses by corroborating our experimental results with independent datasets. This integrative approach strengthens the reliability of our conclusions and contributes to the cumulative knowledge base in the field.

Additionally, we acknowledge that our study may not introduce a novel technique or tool (which wasn’t the scope of this study); however, it significantly enriches the available resources by providing comprehensive spatiotemporal expression profiles for teneurins and latrophilins in the developing mouse brain. As mentioned in the discussions, the Allen Developing Mouse Brain Atlas does not include in-situ data for Tenm and Lphn expression (except Tenm3). By filling this gap, our data serve as a valuable reference for researchers investigating neurodevelopmental processes and synaptic biology. We also would like to clarify that the scope of this work is to provide as a resource rather than a novel technique or tool. In summary, we believe that the detailed spatiotemporal information we have provided, along with the validation of our findings using publicly available datasets, establishes our work as a valuable resource for the neuroscience community.

Reviewer #3: This interesting manuscript from Liakath-Ali et al.  presents a in-situ hybridization mapping of all teneurins (Tenms) and latrophilins (Lphns) in the mouse hippocampus from prenatal (e16.5) to postnatal development (P30). This approach is complemented by the analysis of several public RNA-seq repositories where they have also analyzed another relevant Lphn ligands such as FLRTs. Part of the results described here are generally confirmatory of previous studies done by the same group and others (ex. Tenm3, Lphn1 and Lphn2 in the hippocampus).  The expression pattern of these proteins is very interesting and timely as a resource publication, considering their importance in regulating topographic mapping in different brain regions, for example as shown in the hippocampus for Tenm3. The methods are solid, the in-situ images of high quality and the results are well presented. Nevertheless, I do have some questions and suggestions that should be addressed:

We thank the reviewer providing constructive feedback. We believe the suggested experiments add more strength to the paper.

1)    Page 5, line 22: it is stated that multiple Tenms are co-expressed in the same neurons but there are no high magnification images or quantification at the cellular level provided to showcase it. Given that it has been shown that some Tenms are co-expressed together with different Lphns in the same neurons (for example in cortex during development), authors should provide information in this direction, for example by performing in-situ hybridization combining different Tenms and Lphns probes and taking closer up images.  As a resource paper, it will help to open new hypotheses based on the possible cis vs trans interactions that these proteins could have in different regions of the hippocampus.

We performed new in-situ hybridization experiments on P30 brain sagittal sections with following five combinations:

1. Tenm3+Lphn2+Lphn3
2. Lphn1+Tenm2+Lphn3
3. Lphn1+Lphn2+Tenm1
4. Lphn1+Lphn2+Tenm4
5. Lphn1+Lphn3+Tenm4

The limitation of the channels of in-situ probes to mix and match didn’t allow us to perform combinations other than these five combinations. Tenms and Lphns expression largely overlapped in CA1 s.p cells and DG granule cells, albeit with varying levels of expression. However, in DG mossy cells we observed both overlapping and non-overlapping patterns of Tenm and Lphns expression. For example, Tenm3 and Lphn3 expression in mossy cells doesn’t overlap (Fig. 7B), whereas Lphn1 and Tenm4 largely overlap in these cells (Fig. 7F). These in-situ images are added as a separate main figure (Fig. 7) and discussed our observations.

2)    I found Figure 3 panel E very useful to see the layer-specific expression of Tenms in the hippocampus. It will be great if a similar panel is made for Lphns because all the images of Fig. 4E-G and S12,13 are low magnification, and it is difficult to see their expression in different layers. Another option is to provide a closer up images for Lphns.

We agree. A high-magnification image panel is now added as Fig. 5E for all Lphns expression in CA1 and CA3 for P10 and P30 stages.

3)    Figure 6 panel C show an interesting expression pattern of Lphns in the entorhinal cortex, where Lphn1 seems enriched in upper, Lphn2 in mid and Lphn3 in lower layers. It would be great if authors could provide a quantification to confirm this finding because it could open new hypothesis about their function.

We quantified the intensities of Lphn1-3 ISH signals in EC layers from two ventral brain sections (blue and orange rectangle boxes in Fig. 6B, C). Indeed, we observe a layer-specific enrichment of Lphns. we also note that even within a specific layer, Lphn expression is patchy due to heterogeneity. These data now added as Fig. 6D.

4)  In figure 7, authors asked whether the lateromedial heterogeneity of Tenms and Lphns at P30 correlate with RNA-seq data published from different hippocampal regions of P26-35 mouse (Cembrowski et al. 2016). The correlation is good for Lphns, and in case of Tenms, there are examples where both datasets go in line, such as (1) Tenm1 expression in ventral CA1 (Fig. 7A) correlating with lateral CA1 (Fig. 3A), or (2) Tenm4 in ventral DG (Fig. 7A) with lateral DG (Fig. 3D).  However, in case of the expression of Tenm2 in ventral DG and Tenm4 in ventral CA1 (Fig. 7A), it seems the correlation is less obvious. It will be great if the authors can clarify this point.

This figure is now Figure 8. We agree that Tenm2 in vDG is higher compared to dDG in Hippo-seq datasets, whereas it is lower in lateral DG, a region that we correlated to ventral region. This also true for Tenm4 in vCA1. This discrepancy may be due to specific expression levels of Tenm2 in Rbp4+ cells in granule cells (Hipposeq was performed in Rbp-Cre lines to capture granule cells from both blades of the DG). Likewise, Tenm4 levels in Vipr2+ pyramidal cells of CA1 (Vipr2-Cre line in Hipposeq). We clarified this in the relevant results section. (Page 9, 10).

5)    In figure 7, authors include FLRTs due to their known interaction with Lphns and Tenms and analyze their spatial expression pattern using public available RNA-seq data. Given that FLRTs are also analyzed in Figure 8 with other RNA-seq databases, and recent publications from the same group have shown the relevance of FLRTs for Tenm-Lphn function in the hippocampus. The expression pattern of FLRTs present in Figure 7 should be confirmed by in-situ hybridization. This is important because some results from RNA-seq data seem to be slightly different with respect regular in-situ hybridizations performed in P56 mouse hippocampal sections (de Wit et al., 2014). For example, in that paper FLRT2 is mainly expressed in d FLRT3 in CA3 and DG. Based on Fig. 7C it seems the levels of FLRT3 in CA2 are similar to DG, or in case of the bubble plot (Fig. 8C), the levels of FLRT2 in CA3 or FLRT3 in CA1 seem quite high respect to the in-situ data.

We performed a new set of in-situ experiments using Flrt1, Flrt2 and Flrt3 probes on P30 mouse brain sections. We found that dorsoventral gradient expression of Flrt1 and Flrt2 in CA1 inferred from Hipposeq data largely correlated with our lateromedial in-situ data. Flrt3 expression largely remained smooth in dorsoventral and lateromedial axis, albeit a slight gradient in CA1 and CA3 s.p. layers. These results are now added as Fig. 8E, F. (Page 10)

Our in-situ data shows Flrt2 expression mainly in CA1 and CA2 with a slight expression in CA3. Flrt3 expression is much higher in CA3 compared to CA1 and CA3. Again, the difference in RNA-seq data might arise from the cre-specific approach they used in Hipposeq datasets (See comment #4).

6)    I found the discussion a bit general and with few examples of interesting expression patterns, for example it is mentioned that Tenm1 could be relevant for experience-dependent plasticity in the CA1 region, but other tenms such as Tenm4 and Tenm2 with strong expression and interesting patterns are not mentioned. I think the discussion should integrate findings with what is known about Lphns and Tenms in topographic mapping, and how these findings correlate with the expression pattern and gradients that are found in the in-situ hybridization mapping. Authors could comment on specific interactions between Tenms and Lphns that could be relevant for the hippocampus organization.  

We now expanded the discussion section with information on Tenm3 and Tenm4 expression and function. (Page 14, 15)

Minor comments:
1)    Page 5, line 12: it is stated that Tenm2 and Tenm4 showed similar higher expression levels when compared to Tenm3 (Fig. 2A-D, S2-5). However, to this reviewer it seems the expression of Tenm3 is less respect to Tenm2 or 4 in sagittal sections at P0 (S4, and Fig 2B).

We now corrected this sentence (Page 5).

2)    Page 5, line 13-14: it is stated that Tenm1 is highly expressed in CA1, CA2 and CA3 at P10 and P30, but from the images of S7 and S8 it seems that the expression is much higher in CA1 compared to CA3.

We agree and this has been changed now. (Page 5)

3)    Page 5, line 20-21. It is stated that all Tenms are present in the DG but with reduced levels of Tenm2 and Tenm4 at P30. However, based on the images of Fig. 2E and S8/S9, it seems the levels in the stratum granulare of the DG for Tenm3 are stronlgy reduced respect to Tenm2 and Tenm4.

The intensity level of Tenm3 in DG reflects from its strong expression in some sparse cell populations (might include interneurons). We observe this in the new experiment where we performed combinatorial in-situ for Tenms and Lphns with high-magnification imaging as suggested earlier (see comments #1), Fig. 7B.

4)    I could not find the information about the number of biological replicates that were used to generate the heatmaps and bubble plots for Tenms and Lphns.

We used one mouse per developmental time point, such parameter is common in large-scale studies, especially when the study’s focus is on WT background which inherently reduces biological variability compared to different genetic backgrounds or treatments. Using multiple mice per time point would significantly increase the cost of the study. Therefore using one WT mouse is appropriate for large-scale approaches that we performed in this study. (Page 16)

5)    I have a question about the RNAscope probes that were used, why in case of Tenm4 authors used a custom-made probe and not the regular probe 555491? Lphn1 RNAscope catalog number is not complete and it is missing for Lphn3.

The RNAScope company (Advanced Cell Diagnostics) makes custom probes on request when the probe (like Tenm4) is not available off the shelf and call it as a custom probe when they sell it. However, after six months of purchase they make it available for sale with a catalog number! So, apparently our then custom Tenm4 probe is now sold as #555491. We now changed this in the methods along with corrections for Lphn1 and Lphn3 probe catalog numbers. (Page 16).

6)    Authors stated that a Java-based program was used to threshold the images and quantify the intensity value, is the program used Fiji / ImageJ?
No. We used a custom-built Java-based program to quantify intensity values and register imaged to Allen Brain Mouse Brain Common Coordinate Framework. We intend to publish this tool in a follow-up study where we will include additional features (for all brain regions in both sagittal and coronal sections) and it will be made accessible for the whole community. A beta version is available for download from our lab website - <https://med.stanford.edu/sudhoflab/science-resources/tools.html>
